# Dermatology

**Published:** 1905-08-12

**Authors:** 


### DERMATOLOGY.

*(Continued from page 329.)*

**Pruritus Ani.**—Sir Chas. Ball[6] divides cases of pruritus ani into three classes:—(1) Those due to parasites, either animal or mycotic; (2) Those due to dermatitis of the cutaneous portion of the anal canal and surrounding skin; (3) Those in which the disease is essentially in the nerves supplying the affected areas with sensation. In the third group external applications in many cases prove useless, and the patients undergo the most chronic torture. For the relief of this affection he suggests making an elliptical incision round the anus and undercutting the skin, thereby severing the nerve branches which supply the sensation. The after-effects in three cases so treated are so hopeful as to lead him to advocate its general use. On the analogy of neuralgia and the necessity often found of removing the superior ganglion, he would not hesitate to remove the corresponding posterior roots of the sacral ganglia (third and fourth) supplying the part, but with the relief thereby produced there would be the possibility of causing loss of the sphincter-control.

**Pyroloxin in the Treatment of Skin Disease.**—Jamieson[7] recommends oxidised pyrogallol, Unna's pyroloxin, for intractable eczema palmaris. Unna recommended it as a remedy for psoriasis and leprosy, and more recently in the form of a plaster-muslin with zinc oxide in lupus erythematosus. Jamieson uses it with glycerine of starch and resorcin 15 grs. ad ℥i., and holds that though the resorcin may contribute to the good result obtained, yet it is certain that the oxidised pyrogallol largely contributes. At the same time he extols the treatment by oxidised pyrogallol in psoriasis, which he says stands next to, or even exceeds, that by chrysarobin. It is not so liable to produce dermatitis, and is more lasting in its beneficial results. It acts more slowly than chrysarobin and produces a similar whitish ring round the lesions, but it has less staining effects. It may be used after scales have been removed with soap or salicylic vaseline in the following ways :—In vaseline ℥i., ʒ½ of the pyroloxin with Ac. salicyl gr. x. as a mordant. If the patches are smooth, a varnish may be better made of benzol 20 parts, acetone 80, pyroloxin 10. In indolent psoriasis tar is added to the solution ; five parts of pix liquida or carbonis to five of pyroloxin. He has used it with success in lichen planus, the treatment having greatly reduced the itching and favoured the disappearance of the eruption. In lupus erythematosus it has the disadvantage of leaving a deep black stain in the parts treated. In eczema it may act like a charm, but in some instances aggravates the inflammation. Yet in infantile eczema of the face it may give brilliant results when used in the following way :—The skin is cleansed with repeated poultices of cold borated starch, and the acute stage soothed by applying weak salicylic vaseline, then to the still irritated and reddened surface a thin coating of Lassar's paste containing 10 grs. of pyroloxin is smeared on, and the disease has in not a few instances quickly subsided. He advises pyroloxin as an after-treatment when in staphylococcus sycosis the hairs have been removed by $x$-rays, and also in ringworm of the scalp it has given good results, especially in those conditions of pityriasis capitis to which Sabouraud has drawn attention as arising from sections of the scalp affected by the tinea tonsurans.

**Artificial Cultivation of B. lepræ.**—N. Rudolf[8] claims that he has cultivated B. lepræ in a salt-free meat extract. This extract is prepared by carrying steam through pumice which has previously been impregnated with a mixture containing an ounce of meat extract to 2 ounces of water. The steam is received in a cooled condenser, and the volatile constituents of the meat obtained in this way are free from salt. Two litres are obtained from each ounce of meat. The fluid is then inoculated with B. lepræ, and the flasks are maintained at a temperature of 37° for four to six weeks. Turbidity should be observed. If the fluid is then filtered twice through a Pasteur filter and reduced to $\frac{1}{10}$ of its bulk in vacuo over sulphuric acid, and glycerine added in equal quantity, a fluid is obtained which Rudolf claims has a curative power in leprosy. If 10 c.c. of this "leproline" are injected a violent reaction ensues in leprosy; perspiration returns to dry parts, sensation returns, strength is gained and contractions loosen, ulcerations heal, while nodules and tubercles gradually flatten and disappear. Mental conditions and general health improve remarkably. While some cases so treated are well, all have shown improvement.

6 Brit. Med. Jour., Jan. 21, 1905. 7 Edin. Med. Jour., May. 8 Med. Rec., April 22, 1905.

